# Predicting substrates for orphan solute carrier proteins using multi-omics datasets

**DOI:** 10.1186/s12864-025-11330-5

**Published:** 2025-02-11

**Authors:** Y. Zhang, S. Newstead, P. Sarkies

**Affiliations:** 1 Department of Biochemistry, University of Oxford, Oxford, OX13QU UK; 2 Kavli Institute for Nanoscience Discovery, University of Oxford, Oxford, UK

**Keywords:** Membrane Transport, Solute Carrier Proteins, Deorphanization, Computational Biology, Gene expression, Metabolomics

## Abstract

Solute carriers (SLC) are integral membrane proteins responsible for transporting a wide variety of metabolites, signaling molecules and drugs across cellular membranes. Despite key roles in metabolism, signaling and pharmacology, around one third of SLC proteins are ‘orphans’ whose substrates are unknown. Experimental determination of SLC substrates is technically challenging, given the wide range of possible physiological candidates. Here, we develop a predictive algorithm to identify correlations between SLC expression levels and intracellular metabolite concentrations by leveraging existing cancer multi-omics datasets. Our predictions recovered known SLC-substrate pairs with high sensitivity and specificity compared to simulated random pairs. CRISPR-Cas9 dependency screen data and metabolic pathway adjacency data further improved the performance of our algorithm. In parallel, we combined drug sensitivity data with SLC expression profiles to predict new SLC-drug interactions. Together, we provide a novel bioinformatic pipeline to predict new substrate predictions for SLCs, offering new opportunities to de-orphanise SLCs with important implications for understanding their roles in health and disease.

**Supplementary Information:**

The online version contains supplementary material available at 10.1186/s12864-025-11330-5.

## Introduction

Solute Carriers (SLCs) represent the second largest family of membrane proteins in the human genome after G-protein Coupled Receptors (GPCRs). According to the latest classification database, 456 protein-coding genes are classified as SLCs [1]. SLC proteins are localised throughout the cell, across cell and organelle membranes, and regulate the flux of many different classes of small molecules, including ions, sugars, amino acids, peptides, vitamins, and nucleotides [2, 3].

SLCs are alpha-helical integral membrane proteins and operate under the influence of ion or metabolite gradients to transport molecules across membranes using an alternating access cycle. Although several versions of the alternating access cycle have evolved, they all share the same fundamental process: an outward open state, wherein a central binding site is available to the non-cytoplasmic side of the membrane; an occluded state, where ions and/or metabolites are trapped inside the transporter; and an inward-facing state, where the transporter has opened its binding site to the cytoplasm [4].

The breadth of substrate specificities and subcellular localization of SLCs gives them critical roles in regulating cellular metabolism [5], energy production [6–8], signal transduction [9], and the maintenance of physical characteristics such as cell volume [10]. Inherited mutations in SLCs have been linked to at least 100 monogenic disorders [11]. Beyond their physiological substrates, SLCs also transport drug molecules and are thus of crucial importance in pharmacokinetics, drug sensitivity and therapy outcomes [12–14].

The key roles played by SLCs in regulating cellular metabolism and their potential to control the efficacy of drug treatments mean that the characterisation of both physiological and non-physiological substrates of SLCs is of great importance to the pharmaceutical industry. However, many SLCs have no substrate annotated and are described as ‘Orphan Transporters’. A recent survey reported that around 28% of SLCs had no experimentally determined substrate [3]. The key challenge to de-orphanising SLCs is the inability to predict substrates based on sequence or structure alone, as many transporters within the same family recognise chemically diverse molecules [1]. For example, members of the SLC13 family share 40%—50% amino acid identity but separate into two different groups based on function: whilst SLC13A1 and SLC13A4 transport sulfates, SLC13A2, SLC13A3 and SLC13A5 recognise carboxylates [15]. Therefore, it is currently not possible to accurately infer SLC substrates from their amino acid sequence, and experimental determination of substrates is required. However, screening enough substrates to cover all the possibilities is not practical. Thus, methods that could produce predictions for the likely substrate of any given SLC would be extremely valuable in narrowing down the list of substrates to test in functional assays, as well as suggesting insights into cellular functions of uncharacterised SLCs.

Here, we describe a new method to use existing multi-omics high throughput datasets to predict SLC substrates. We demonstrate that our method carries predictive power in recovering known SLC-substrate pairs when applied to multiple major cell line panels. We use our method to generate predictions for orphan SLCs. In parallel, we develop our method to produce new predictions for the effects of SLC expression on sensitivity to drugs. We hope that our predictions act to generate new hypotheses for the substrates, cellular roles, and therapeutic implications for uncharacterised SLC proteins.

## Results

### Correlation analysis between metabolomics and expression datasets successfully predicts SLC substrates

**Fig. 1:**
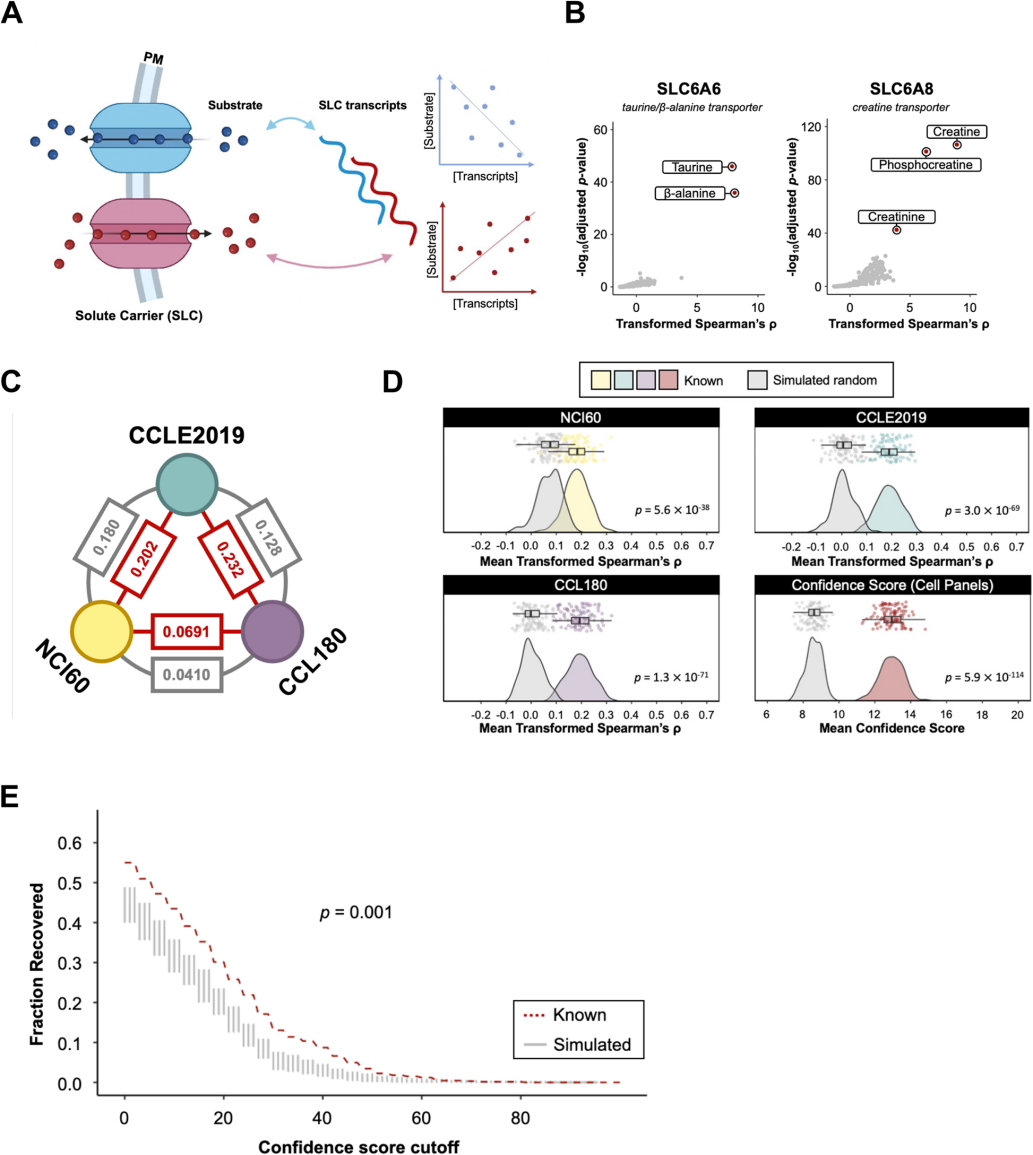
Correlating SLC transcript levels to substrate concentrations reveals known substrates of SLC. **A** Schematic representation shows the principle of correlation analysis. SLC expression levels are hypothesised to correlate with the intracellular concentration of their corresponding substrates. Blue, SLC exporting substrate; red, SLC importing substrate. Figure created with elements from BioRender. **B** Scatter plot shows transformed Spearman’s ρ and adjusted *p*-value for 225 metabolites correlating with the expression level of SLC6A6 and SLC6A8 across 913 cell lines from CCLE. Key substrates are labelled. **C** Mutual concordance of SLC-metabolite correlation outcomes from different datasets, each processed with the pipeline specified in Methods. Nodes, datasets, edge, concordance parameter measured in Spearman’s ρ. Dark red represents the concordance are assessed in the known correlation pairs, while grey represents the concordance are assessed in all correlation pairs. CCLE2019 and NCI60, *p* = 4.30 × 10^–293^ (all), *p* = 1.60 × 10^–4^ (known); CCLE2019 and CCL180, *p* = 1.50 × 10^–221^ (all), *p* = 6.56 × 10^–221^ (known); NCI60 and CCL180, *p* = 2.38 × 10^–24^ (all), *p* = 0.197 (known). **D** Distribution plots show the distributions of 100 bootstrapped mean transformed Spearman’s ρ or mean confidence score in known SLC-metabolite set compared to 100 simulated random sets in each dataset. Colored distribution curves in yellow (top left), green (top right), and purple (bottom left) represent the bootstrapped mean transformed Spearman’s ρ in NCI60, CCLE2019, and CCL180, respectively; Colored distribution curves in dark red (bottom right) represents the bootstrapped confidence score calculated from evaluation of correlation strength of the 3 previous datasets; grey distribution curves represent respective simulated random sets that were generated independently for each datasets. *p*-value was derived from one-tailed t-test against the null hypothesis that mean transformed Spearman’s ρ (or confidence score) of known set is not higher than its corresponding 100 simulated random sets. **E** Across confidence score cutoff selected (from 1 to 100), fraction of pairs recovered (having confidence score better than cutoff) in known set compared to in simulated random set. Red dashed line, recovered fraction value in the known set; grey violin distribution recovered fraction value in the simulated random set; *p*-value was derived from one-tailed Wilcoxon test against the null hypothesis that fractions recovered in the known set are not higher than its corresponding 100 simulated random sets

We set out to de-orphanise SLC proteins by investigating the potential effects their expression might have on cellular metabolite concentrations. We reasoned that the transporter activity of SLCs might result in correlations between SLC expression level and the intracellular concentrations of their corresponding substrates (Fig. 1A). We used a major cancer cell panel profiling 225 metabolites with liquid chromatography-mass spectrometry (LC–MS) across almost a thousand cancer cell lines from the Cancer Cell Line Encyclopedia 2019 (CCLE2019) [16]. We selected a list of 447 SLC and 72 SLC-like genes (S1 Table) from previous curations [3, 17]. For each SLC or SLC-like gene in the list, we obtained Spearman’s rank correlation coefficients (ρ) between normalised transcript levels and metabolite concentrations in 913 cell lines and calculated the Z-score of the absolute values of the correlation coefficients (transformed Spearman’s ρ) for each metabolite to account for varying degrees of correlation strength across different metabolites (see Methods). Upon inspection of this set, we observed many cases where the expression of an SLC correlated most strongly with its known substrate. For example, SLC6A6, a Na^+^/Cl^−^-dependent β-alanine and taurine transporter [18], correlated most strongly to β-alanine and taurine, whilst SLC6A8, a Na^+^/Cl^−^-dependent creatine transporter [19], correlated most strongly to creatine (Fig. 1B). Notably, the expression of SLC6A8 also strongly correlated with two other metabolites, phosphocreatine and creatinine, which are direct derivatives of creatine [20, 21].

These examples indicated that the expression and metabolite level variation across cancer cell lines might be generally predictive of the functions of SLCs. To test this systematically, we expanded the correlation analysis to two other major cell line panels, NCI-60 [22] and CCL180 [23] (Table S2-S4), and updated the database of SLC annotations based on previous report [3], forming a list of 667 known transport activities with substrates or substrate derivatives present in the metabolomics datasets (Table S5; see also Table S1 for references). Since the annotated names for the same metabolites were often different across the databases, we manually examined 1980 metabolomic annotations in the three metabolomics we used to ensure consistent nomenclature (Table S6). Next, we assessed the concordance for all correlation pairs among the three analyses with Spearman’s correlation (Fig. 1C). We noticed that the concordances in the known pairs are stronger compared to all pairs among datasets, demonstrating consistency across datasets. However, the relationship is rather weak for CCL180 and NCI60, regardless of which pairs were assessed. The low concordance might be due to the different cancer types used and different methodologies in measurements of metabolite levels [23, 24].

We were curious as to whether the transformed Spearman’s ρ between SLC-substrate pairs were better than randomly permutated pairs of the same set (“simulated random pairs”). We bootstrapped 100 mean-transformed Spearman’s ρ out of 667 known pairs (excluding all derivatives) and compared them to 100 mean-transformed Spearman’s ρ from 100 iterations of 667 simulated random pairs. All three datasets showed significantly higher mean transformed Spearman’s ρ values (Fig. 1D). It should be noted that the NCI60 dataset demonstrated a tendency for random pairs to be higher than the other two datasets, representing a systematic bias within this dataset specifically. However, the true pairs still significantly outperformed random pairs in this dataset (Fig. 1D).

Datasets, although different, captured correlations from transport activities. We reasoned that novel SLC-substrate pairs might become apparent if we unify the datasets. Therefore, we defined a confidence score as a measure of the correlation strength across the three datasets. The confidence score was calculated for every pair per dataset by assessing whether its transformed Spearman’s ρ was higher than the dataset-specific lower bound (“Threshold of Discovery”) we set and how it ranked among the known pairs in this dataset. A threshold of discovery of 0 means that every pair with transformed Spearman’s ρ lower than 0 (i.e. the Spearman’s ρ is lower than the mean among the correlation between this SLC and all other metabolites) will be assigned a confidence score of 0, while if higher, the confidence score will be from 1 to 11 according to the ranking relative to known SLC-substrate pairs. The threshold of discovery was chosen to maximise the fractional difference between true positive (fraction of the known pairs with a higher score than the cutoff) and false positive (100 fractions of the simulated random pairs with a higher score than the cutoff) across a range of confidence score cutoffs in the dataset (Figure S1A-C). A final confidence score for the pair will thus be the sum of the three from each dataset (see **Methods** Confidence score). We demonstrated that the mean confidence scores for the known SLC-substrate pairs are significantly higher (Fig. 1D), and the fractions recovered in the known set across a range of confidence score cutoffs are consistently higher (Fig. 1E; Figure S3A, S3B; ROC AUC = 0.5704, PR AUC = 0.5880), compared to simulated random pairs. Taken together, these results indicate that correlation analysis can detect SLC-substrate pairs, and the unification of correlation strength from different datasets could be a potential method to predict new substrates for orphan SLCs.

### Data from gene dependency screens improves SLC substrate predictions

**Fig. 2:**
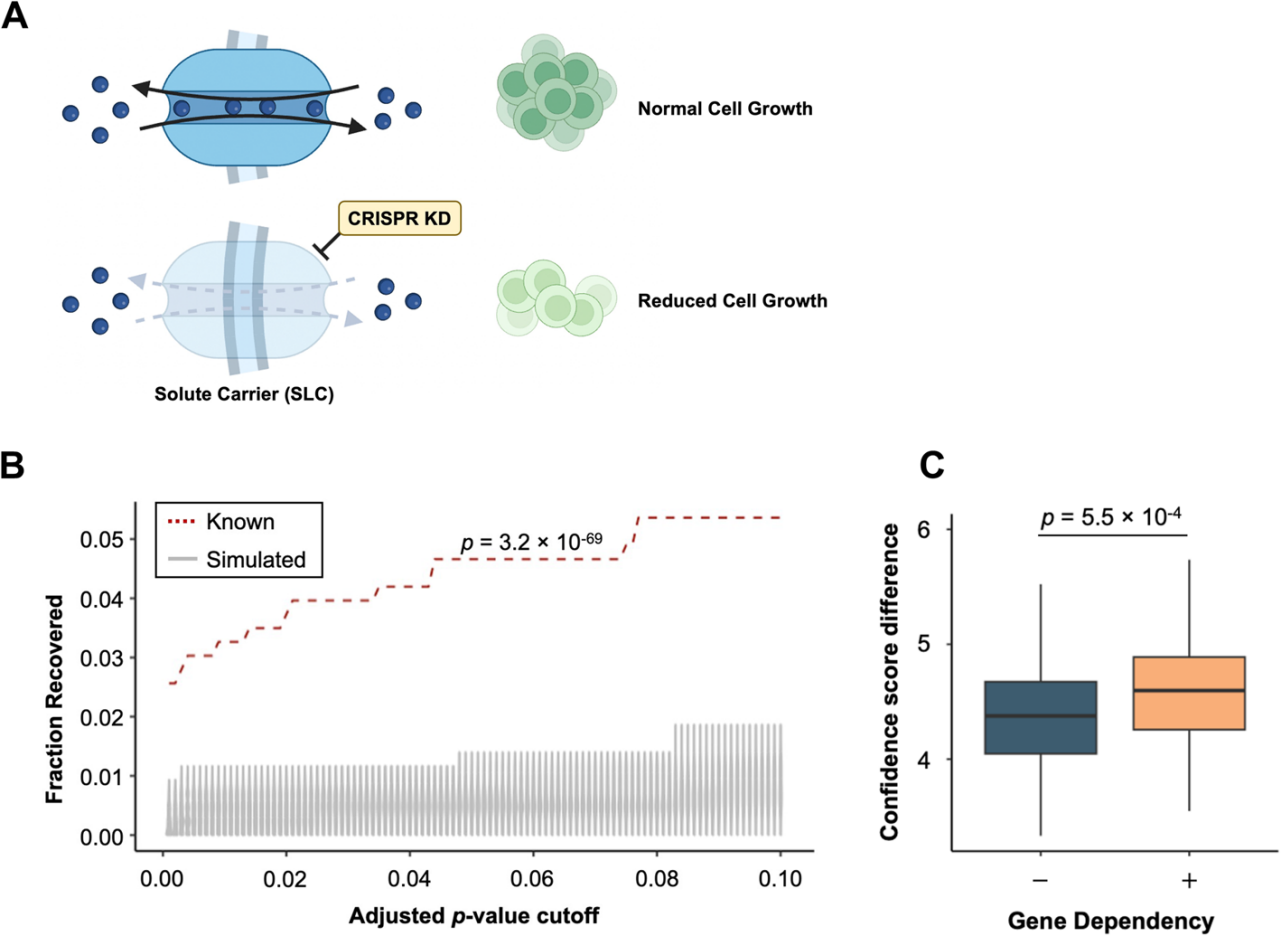
CRISPR loss-of-function screen provides alternative source for SLC prediction. **A** Schematic representation shows the principle of CRISPR loss-of-function data analysis to predict SLC substrates. Figure created with elements from BioRender. **B** Violin plot shows the recovered fractions in known sets compared to simulated random sets across different cutoff selected for resulting adjusted *p*-values. The *p*-value comparing the fraction distribution between known and simulated random was derived from one-tailed Wilcoxon test against the null hypothesis that fractions recovered in known set is not higher than its corresponding 100 simulated random sets. **C** Boxplot showing the effect of incorporation of CRISPR loss-of-function screen into prediction algorithm on the confidence score difference between true positive and false positives (“$+$”), compared to considering cell panels alone (“$-$”). The *p*-value comparing the confidence score difference was derived from one-tailed Wilcoxon test against the null hypothesis that confidence score difference calculated using both correlation analysis and CRISPR loss-of-function screen (“$+$”) is not better than only using correlation analysis (“$-$”)

We next considered whether data from genome-wide gene dependency screens [25] could be incorporated into our predictions of SLC substrates. We reasoned that cell growth may be dependent on a specific SLC if the cells grow more slowly after the expression of that SLC is depleted due to the loss of that particular metabolite(s) (Fig. 2A). Metabolites whose concentrations are significantly different between dependent and non-dependent cell lines might, therefore, be candidate substrates for the SLC in question. We used the CRISPR-Cas9 dependency screen [26], recording cell growth in over a thousand cell lines upon CRISPR knockdown, of which 625 cell lines overlap with CCLE2019 metabolomics profiling. To infer the dependency of cell lines on specific genes, we used the gene effect score, which had been defined previously [27]. Genes annotated with negative gene effect scores indicated that cells exhibit reduced proliferation upon their deletion compared to normal cells. For each SLC gene in the curated list, we ranked the cell lines based on their corresponding gene effect scores, excluding positive scores where growth was improved by loss of the SLC. We computed a *p*-value following multiple test corrections for the difference in each metabolite between cell lines with the top 20% of negative scores (more dependent) and bottom 20% of negative scores (less dependent) (Table S7). Across a range of *p*-value cutoffs, the fraction of significant pairs recovered from the known set is higher than that of simulated random pairs. The fractional difference is maximised when the *p*-value cutoff for significance is set to 0.16 (Figure S1D). For *p*-values smaller than the cutoff, a confidence score is assigned based on its position within a similarly calculated decile-based quantile of known pairs, scaled to 1.0 (Figure S1E; see **Methods** Confidence score). Thus, incorporating the CRISPR-Cas9 dependency screen as an additional data source improved the prediction performance, as the confidence score difference between known pairs and simulated random pairs increased by 5% (Fig. 2C), as well as a slight improvement in the ROC and PR curve (Figure S3A, S3B; ROC AUC = 0.5723, PR AUC = 0.5920).

### Inclusion of adjacent metabolites improves substrate prediction for SLCs

**Fig. 3:**
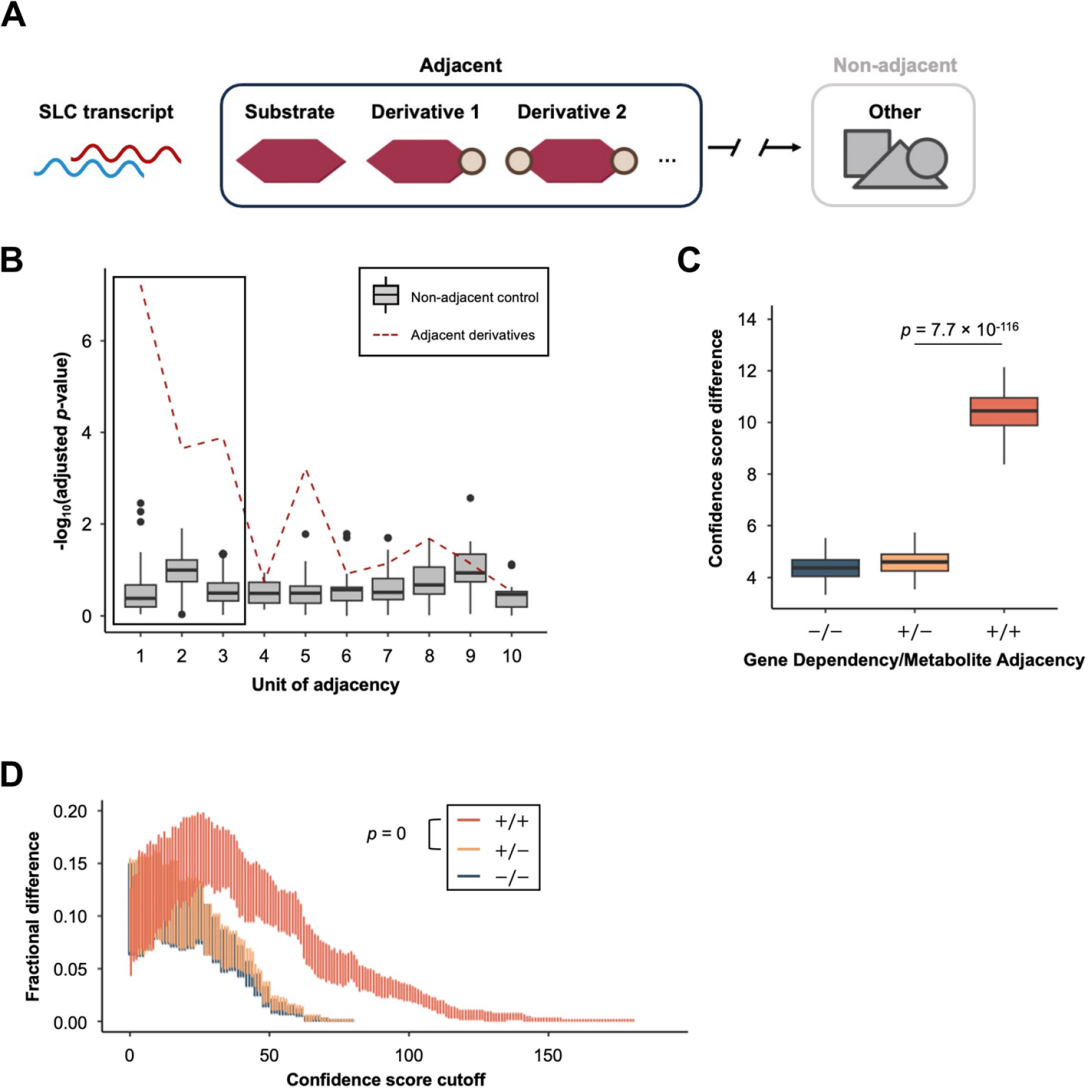
Inclusion of metabolite adjacency to the prediction substantially differentiates known interaction from simulated random interaction. **A** Schematic representation shows the relationship between adjacent metabolites (*i.e.*, derivatives of the substrate) and non-adjacent metabolites (*i.e.*, not derivatives of the substrate). Pipeline details specified in Method. Figure created with elements from BioRender. **B** Boxplot shows the general similarity of Spearman’s ρ between derivative across unit of adjacency. Red dashed line, Spearman’s ρ differences between adjacent derivatives and substrates are compared to non-adjacent controls across unit of adjacency. Grey boxes, Spearman’s ρ differences between non-adjacent controls are compared to another 100 non-adjacent controls across unit of adjacency. Black rectangular highlights the selectable range of the unit of adjacency (1, 2, 3). **C** Boxplot shows the effect of the incorporation of metabolite adjacency into the prediction algorithm ($-/-$, cell panels; $+/-$, cell panels and gene dependency; $+/+$, cell panels, gene dependency, and metabolite adjacency). The *p*-value comparing the confidence score difference was derived from one-tailed Wilcoxon test against the null hypothesis that confidence score difference calculated adding metabolite adjacency (“$+/+$”) is not better than only using correlation analysis (“$+/-$”). **D** Violin plot shows the fractional difference between true positive and false positives calculated with the inclusion of metabolite adjacency compared to the algorithm without including metabolite adjacency over a range of confidence score cutoffs ($-/-$, cell panels; $+/-$, cell panels and gene dependency; $+/+$, cell panels, gene dependency, and metabolite adjacency). The *p*-value comparing the confidence score difference was derived from one-tailed Wilcoxon test against the null hypothesis that confidence score difference calculated adding metabolite adjacency (“$+/+$”) is not better than only using correlation analysis (“$+/-$”)

Our results demonstrated that the correlation analysis and CRISPR-Cas9 dependency carry predictive power towards recovering known SLC-substrate pairs (Figs. 1 and 2). However, substrates might easily dissipate into downstream derivatives, leading to poor correlation and reduced predictive power. Furthermore, the prediction of substrates will be reinforced if the SLC correlates with the derivatives of the substrates as well. To address these ideas, we created a metabolite adjacency matrix (Table S8) from annotated KEGG metabolic pathways. This was done by extracting the number of conversion steps required for one metabolite node to reach another, with each unit of adjacency representing a conversion edge between two metabolite nodes. We reasoned that the expression of the SLC that transports the substrate molecule may correlate with its proximal derivatives, while for its distant derivatives, the distribution of correlation strength will be more random and thus more similar to metabolites that are not related to the substrate of the SLC (Fig. 3A). To validate this hypothesis, we generated adjacency tables containing the derivatives that represent different steps of conversion away from the original SLC-substrate table, from proximal to distant. For each SLC-derivative pair in the table, we measured the similarity between the correlation of SLC-derivative pairs and the original SLC-substrate pairs by calculating their Spearman’s ρ difference. Subsequently, these differences were compared against the control tables containing randomly sampled non-adjacent metabolites. Non-adjacent metabolites cannot be linked to the substrate node via any continuous path. For each non-adjacent control in the previous comparison, 100 non-adjacent controls were compared in a one-to-one manner to ensure robustness. Our results demonstrate that proximal derivatives (those requiring fewer conversion steps) showed greater similarity to the original substrates compared to distant derivatives and that the difference between derivatives and non-adjacent molecules decreased with increasing distance from the substrate, especially for derivatives that are within 3 units of adjacency, regardless of whether the Spearman’s ρ is raw (Fig. 3B) or transformed (Figure S2A). We selected 2 as the unit of adjacency to use for our analyses since this selection produced the highest fractional difference (Figure S2B). We next determined the optimal threshold of discovery (Figure S1F-G) and the confidence score to be added for each discovery (Figure S1I) to maximise the difference between known SLC-metabolite pairs and simulated random pairs (see **Methods** Confidence score). This improved the confidence score difference by 127% (Fig. 3C), fractional difference by 47% (Fig. 3D) and AUC in the ROC and PR curve (Figure S3A, S3B; ROC AUC = 0.5998, PR AUC = 0.6126), indicating that metabolite adjacency information bolstered the accuracy of our prediction algorithm.

### Predicted substrates for Orphan SLCs

**Fig. 4:**
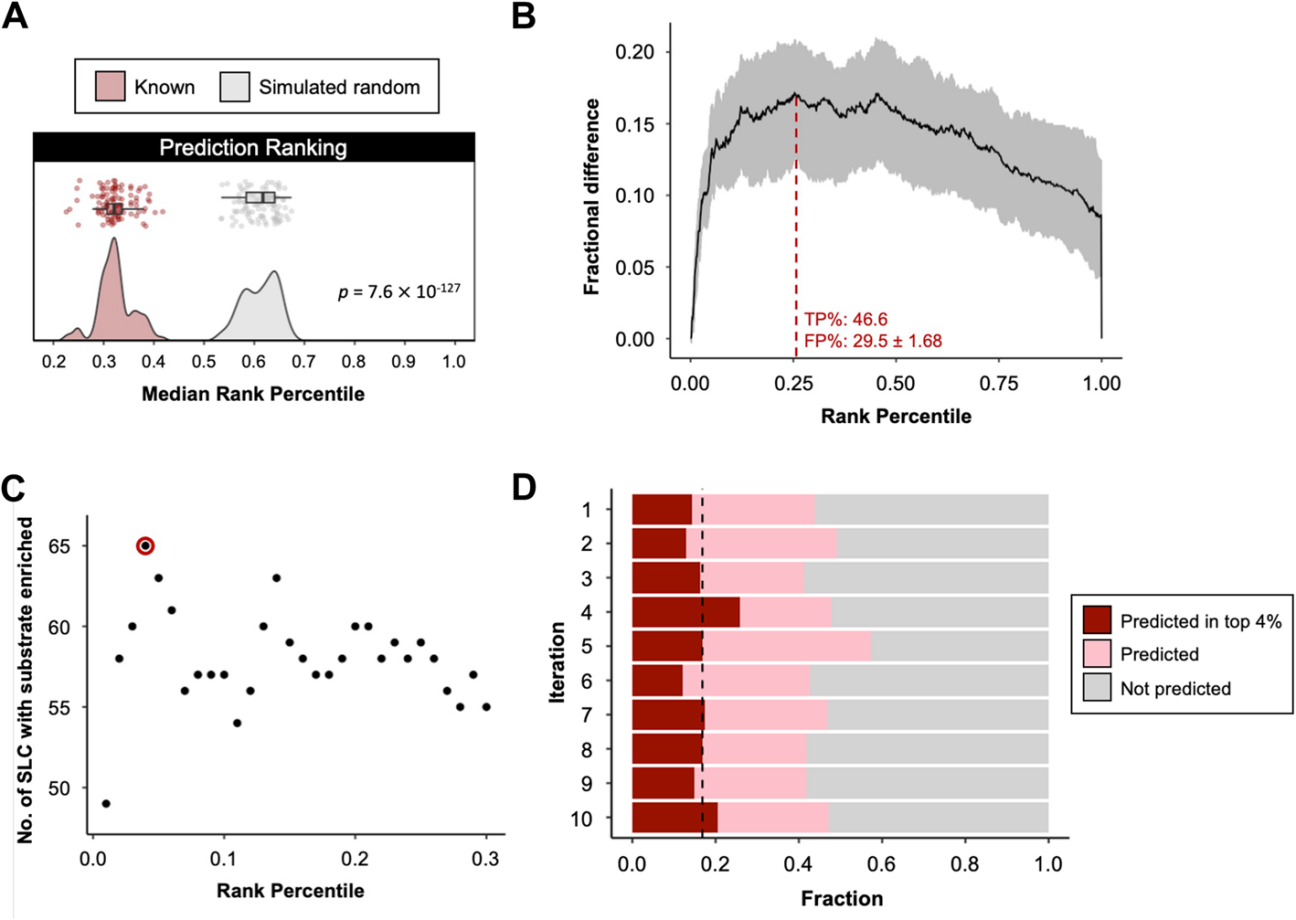
Top-ranking predictions of known transport activity.**A** Distribution plot shows 100 bootstrapped median ranks of known SLC-substrate pairs compared to 100 median ranks in simulated random pairs. The *p*-value comparing the median rank was derived from one-tailed Wilcoxon test against the null hypothesis that the median rank in the known set is not closer to top than in simulated random set. **B** Fractional difference between true positive (TP%) and false positive (FP%) when only rank percentile above the given value is considered as predicted. Black line, median fractional difference; grey, range of fractional difference. **C** Point plot shows the number of SLC with substrate converged with the pathway enriched from metabolites ranked above the given rank percentile in the prediction list. **D** Stacked bar plot shows the fraction of all pairs in the testing set that was recovered in each iteration. Dark red stack, the fractions of pairs in the testing set appeared in the top 4% of predictions per SLC, ranked by confidence scores; pink stack, the fractions of pairs in the testing set with confidence score above locally optimised score cutoff; grey stack, the fractions of pairs in the testing set with confidence score below locally optimised score cutoff. Black dashed line represents the mean fractions of pairs in the testing set appeared in the top 4% across 10 iterations

The previous analysis confirmed that true SLC-substrate pairs tend to associate with higher confidence scores, and increasing confidence scores yielded consistently higher fractions in the known set compared to simulated random. We validated that true SLC-substrate pairs tend to appear in a higher position compared to simulated random pairs when pairs for each SLC were ranked according to their confidence scores, measured with the median rank of the sets (Fig. 4A). We determined that the fractional difference peaked at positive recovery of 46.6% if we only considered predictions ranked within 25% of all metabolites with non-zero confidence scores (Fig. 4B). However, to generate a number of predictions for orphan SLCs that could be reasonably tested experimentally, we sought to reduce the number of predictions further. We reasoned that we could improve the predictive power for a smaller number of possible substrates by simultaneously identifying over-represented metabolite pathways within the set. We curated a list of 623 metabolites across the three metabolomics datasets that could be linked to 57 metabolic pathways (Table S9). Using the known SLC-metabolite pairs, we showed that considering metabolites that ranked in the top 4% of predictions with non-zero confidence scores were optimal to successfully predict enriched metabolic pathways containing the known substrate (Fig. 4C). To further evaluate the prediction method, we ran 10 iterations of partition, training and testing processes (see Methods) using 70% of randomly selected known pairs (training sets) to obtain parameters for the confidence score formula, then using this to calculate scores for the remaining 30% of known pairs (testing sets). Across 10 iterations, the mean confidence scores of training sets are consistently higher compared to simulated random pairs of the training sets (Figure S4). On average, our method robustly predicted 46% of all pairs in testing sets, with almost 17% in the sets appearing in the top 4% among predictions (Fig. 4D; ROC AUC, mean = 0.595, SE = 0.00271; PR AUC, mean = 0.601, SE = 0.00311).

On this basis, we used our prediction algorithm to create a list of substrate predictions with high confidence scores for 128 orphan SLCs at the cutoff of the top 4% per SLC (Table S10). We identified many predictions that are in line with experimental data. For example, we found strong associations between the orphan SLC CLN3 and several glycerol phosphate-related metabolites (e.g. phosphatidylcholine, alpha-glycerophosphocholine, alpha-glycerophosphate, glyceryl phosphoryl ethanolamine), agreeing with recent research indicating that CLN3 mutant in zebrafish leads to glycerophosphodiesters (GPDs) accumulation in early development [28]. We predicted that MTCH1 could be associated with metabolites involved in glutathione synthesis (glycine, glutathione, glutamate, pyroglutamate, NADPH), which aligns with the recent observation that MTCH1-deficiency correlates with NAD^+^ depletion in mitochondria [29]. Moreover, our results converge with a previous attempt to predict SLC substrate predictions that used sequence information [3]. In this publication, SLC25A45, SLC22A25 and SLC35E2B were all predicted to have nucleobase-containing substrates, and our algorithm also predicted a variety of nucleobases as substrates for these transporters (Table S10). Together, our predictions could be used to generate plausible hypotheses for novel SLC substrates, which can be used to narrow down subsets of metabolites for downstream experimental verification and lead to faster de-orphanisation.

### Leveraging drug repurposing panels may predict new interactions between SLCs and cytotoxic drugs

**Fig. 5:**
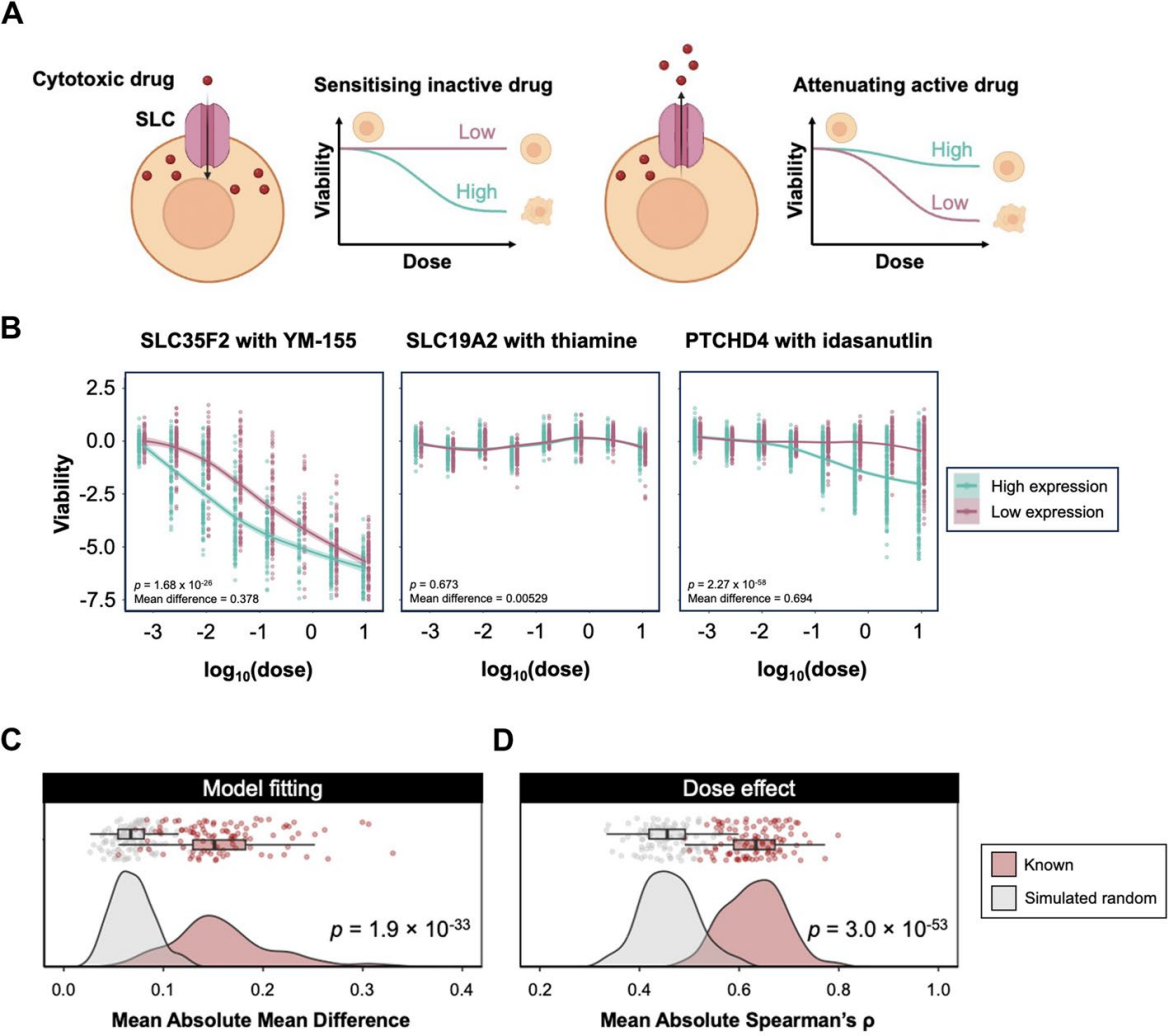
Combining drug sensitivities and SLC expression profile reveals valuable associations between SLC and drug efficacy. **A** Schematic representation shows the principle of leveraging the drug dose response curve to predict SLC-drug associations. If a cytotoxic drug compound is transported by a SLC, expression of the SLC may either enhance or attenuate its killing effect. Figure created with elements from BioRender. **B** Non-linear regression shows an example of how SLC35F2 expression affects the killing efficacy of the known substrate drug YM-155 (Left); an example of how SLC19A2 expression does not affect sensitivity to thiamine (Middle); a prediction example of idasanutlin efficacy might associate with PTCHD4, which is not an identified link. Statistics were computed based on paired t-test of model prediction capturing the shape of fitted regression. **C** Distribution plot shows the distribution of mean absolute mean difference in 100 bootstrapped known interaction sets and in 100 simulated random interaction sets. *p*-value is derived from one-tailed Wilcoxon test against a null distribution that mean absolute mean difference of the known set is not better than those of the simulated random. **D** Distribution plot shows the distribution of mean absolute Spearman’s ρ in 100 bootstrapped known interaction sets and in 100 simulated random interaction sets. *p*-value is derived from one-tailed Wilcoxon test against a null distribution that mean absolute Spearman’s ρ of the known set is not better than those of the simulated random

Solute carriers are known to play an important role in determining drug pharmacokinetics, safety and efficacy profiles [30]. A key goal of the International Transporter Consortium is to identify transporters involved in drug transport and highlight potential issues around adverse drug-drug interactions involving transporters during clinical trials [31]. Therefore, in parallel to the prediction of physiologically relevant substrates, we investigated whether interrogation of omics datasets could be used to identify drug molecules that are substrates for specific SLC proteins. We reasoned that expression of SLCs might affect drug efficacy, thus altering the shape of the dose–response curve reporting the relationship between viability and drug concentration. For example, when considering cytotoxic drugs, if cell death is improved or attenuated with higher SLC expression levels, one possible indication is that the drug is a substrate for transport by the SLC in question (Fig. 5A). We investigated our hypothesis using the cancer repurposing screen profiling 1448 active drugs against 578 cancer cell lines across 8 doses [32]. 569 cell lines were found in CCLE2019 transcriptomics, where we ranked the cell lines according to the expression level of each SLC in descending order. For any SLC, the first and last 20% of cell lines were marked with “high expression” and “low expression”, respectively. The dose–response of cells post-drug treatment annotated with SLC expression level was represented as we fitted local polynomial regression models to the data points. The curves were compared as we calculated predicted viabilities at each predicted dose for each expression annotation and applied a pairwise t-test to derive *p*-values and the absolute values of the mean difference. Our data showed consistency with previously validated results. For example, SLC35F2 expression sensitised cells to the drug YM-155, a known substrate imported by this SLC [14]. SLC19A2 encodes a plasma membrane thiamine transporter [33], but thiamine uptake is not a dose-dependent factor impacting cell viability (Fig. 5B).

To test if the analysis shows systematic power in predicting known transport activity with cellular viability, we curated a list of 180 known transport activities by SLCs on drug compounds (Table S11). We selected 21 activities that satisfied the following criteria: (1) the compound is found in the list of active drugs in the screen; (2) the compound does not target any specific mutation; and (3) a significant difference (*p* < 0.05) in cellular viability is observed between the curves. Benchmarking with 100 simulated random pairs demonstrated that known transport activities correlated with larger viability differences (Fig. 5C) and that viability differences tended to correlate stronger with dosage administered for pairs with known activities, demonstrating dose-dependent effects (Fig. 5D). Both predictors, the absolute mean difference in viabilities and dose-dependent effect, presented good sensitivity and specificity (Figure S3C, S3D; Absolute mean difference, ROC AUC = 0.7080, PR AUC = 0.6814; dose-dependent effect, ROC AUC = 0.6689, PR AUC = 0.6628).

The treatments of cytotoxic drugs to cells create stress and might lead to variabilities in cellular viabilities that are specific to factors other than a particular SLC expression. To test this, for every drug, instead of selecting top and bottom 20% cell lines, we selected two groups of random cell lines, each containing 20% of the total cell line number, and fitted local polynomial regression models to compare the predicted viability difference at each dosage. We repeated this process 100 times and calculated the mean and standard deviation from the 100 log10-transformed *p*-values and absolute mean differences that resulted. A predicted interaction between drug and SLC will only be included if both of their t-test results, *p*-value and absolute mean difference, are at least two standard deviations higher than the mean derived from 100 random cell line picking iterations (Table S12). We selected the top 50 predictions after filtering, ranked with absolute mean difference and dose-dependent effect (Table S13). The SLC-drug pair with the best prediction statistics was an experimentally validated transport activity of YM-155 by SLC35F2 [14]. Our algorithm also predicted previously unknown links; for example, we predicted an interaction between the orphan SLC Patched Domain Containing 4 (PTCHD4) and the drug molecule idasanutlin, which acts as a small molecule antagonist of p53 activity suppressor Mouse double minute 2 homolog (MDM2) (Fig. 5B; [34]). We also noticed a group of SLCs (SLC3A1, SLC7A7, SLC16A4, SLC23A1, SLC37A1, SLC37A2, SLC41A2, NPC1L1, CLN3) that interact with the small molecule inhibitor RITA, which leads to induction of cell apoptosis by (re)activating wild-type or mutant p53 [35]. SLC3A1 is associated with attenuation, while the other is associated with sensitisation of the killing effect (Figure S5A). Importantly, the cell lines exhibiting high and low expression of each SLC were largely non-overlapping, indicating that the sensitivity is likely due to the SLC expression rather than the identity of cell lines (Figure S5B). In summary, our work provided a possible route to predict SLC-drug interactions in parallel to physiological substrate determination, aiding the process of exploring SLC as a therapeutic target reservoir or alerting drug discovery teams to potential downstream issues with cell toxicity or adverse impacts on drug pharmacokinetics.

## Discussion

The deorphanisation of SLCs has been a great collective effort in the community [36–39]. However, experimental substrate determination is hindered by the technical difficulties in expressing and purifying functional membrane proteins and the huge range of potential compounds that could be tested even if the SLC is isolated for functional study. Therefore, accurate prediction of SLC substrates would be an important development for the field. Here we developed a new method to predict SLC substrates and demonstrated we could recover known interactions, indicating its potential for de-orphanising SLCs in the human genome. Below, we discuss how the algorithm compares to previous methods, its strengths and limitations, and prospects for future improvement.

An obvious approach to attempt to predict SLC substrates is to utilise structures of transporters with known substrates to generate a set of rules that could be subsequently used to predict substrates for orphan SLCs. This approach was attempted by [NO_PRINTED_FORM] [3], who trained a machine-learning algorithm with systematic SLC substrate annotations and structural features, such as sequence and topological domains. Probabilities were produced for 115 orphan SLCs against 18 selected substrate terms. Over the subsequent 3 years, substrates have been experimentally defined for 28 of these orphan SLCs, offering the opportunity to evaluate this method (Table S14). Prediction of the substrates of 4 SLCs aligned with experimental results (SLC39A11, TMEM165, SLC16A6, SLC16A17). For example, TMEM165, now characterised as a lysosomal Ca^2+^ importer [40], was predicted to have a high probability of transporting divalent metal cations. SLC6A17 was demonstrated to transport glutamine in mice synaptic vesicles (Jia et al., 2023) and was predicted to transport L-amino acids. However, for the remainder of SLCs where substrates were determined, the algorithm either did not provide any predictions (16/28), or the predictions produced did not match experimental determination completely (8/28). For example, ANKH was predicted to have a high probability of transporting metal ions but turned out to mediate ATP and citrate export [41]. This evaluation showed that the prediction based on structural features has limitations.

In comparison, our method is deliberately agnostic of SLC structural information and instead focuses on how varying levels of SLC expression affect metabolite levels in cells. This method is conceptually similar to genome-wide association studies (GWAS), which link genetic variations, specifically single nucleotide polymorphisms (SNPs), in transporters to changes in the levels of endogenous metabolites [42]. However, the sensitivity of leveraging GWAS is limited as it relies on common SNPs linked to an SLC for it to be interrogated, and of those SNPs that are linked, not all of them may result in alterations in SLC function or expression. Our approach is also agnostic about known biochemical properties, which means that it has the potential to predict surprising or unexpected interactions, even if an experimentally defined substrate has been annotated. This could be important as many substrates are defined experimentally using a limited range of compounds and in vitro assays, which, therefore, may not capture the full spectrum of activities for an SLC in vivo. For example, inositol was found to have a high degree of correlation with the recently characterised facilitative taurine transporter SLC16A6 [43] in all three datasets but not with other SLC16 family members, potentially indicating that this may be an additional substrate of SLC16A6.

The biochemical naivety of our model also presents limitations. The availability of transcriptomics and metabolomics in well-curated cell lines is limited to a small number of datasets comprising cancer cell lines. The rewiring of metabolic pathways has been long established as a hallmark of cancer [44], meaning that some of the associations may be cancer-specific. Nevertheless, this could provide an opportunity for our method to generate information relevant to understanding cancer cell physiology. Another limitation is that correlations between SLCs and metabolites may arise as a result of co-expression with other SLCs. Equally, there are many reasons why the expression of a particular SLC might not correlate with levels of its substrate, for example, post-translational regulation of transport activity, subcellular compartmentalization of the metabolite, transporter redundancy or compensatory effects. Furthermore, our method is limited to metabolites measured robustly in metabolomics experiments. A notable absence from these measurements are most small ions, which are frequently substrates for SLCs [45]. To address the limited metabolic space in our prediction, we included the adjacency information (Fig. 3) for all 1980 metabolites. Therefore, the predicted substrates should be considered as representing a group of potential substrates rather than a specific, clearly defined one. We recommend viewing our method as a tool for generating hypotheses that require further validation through in vitro experiments with purified proteins.

In addition to identifying physiological substrates for SLCs, we also used omics data to identify potential drugs that might be substrates. Previous efforts to predict SLCs targeted by drugs include rational drug design targeting SLC structures, exploring compounds with chemical similarity to known substrates, and discovering inhibitors using in silico screening of ultra-large compound libraries [46]. However, it is important to note that such discoveries are based heavily on high quality biochemical and 3D structural information. Taking protein–ligand docking as an example, despite the immense success of AlphaFold2 and 3 [47, 48], slight differences in the predicted binding site can lead to large inaccuracy in ligand binding [49]. Here, our method presents an alternative and explores the possibility of predicting SLC–cytotoxic drug interaction through cell viability data. A previous experimental approach screened the impact of knocking out specific SLCs on 60 representative cytotoxic drugs, highlighting the broad role of SLCs in drug efficacy [50]. Of the 201 prominent associations (47 drugs, 101 SLCs) they reported, 39 drugs and 97 SLCs are also included in our analysis. Two-thirds (26/39) of these drugs were found to have associated SLCs predicted in our study, with five associated SLCs ranked highly in our predictions: SLC1A4 & Triptolide (3rd out of 92), SLC19A1 & Methotrexate (6th out of 72), SLC2A1 & Idarubicin (6th out of 58), SLC15A1 & 6-Mercaptopurine (7th out of 61), and SLC12A4 & 5-Azacitidine (11th out of 86). The remaining 13 drug associations were eliminated as they fell outside the range of drug-specific thresholds; however, half of these would have associated SLCs found in the top 10% of predictions for each drug, indicating concordance and predictive power.

Our analysis complements this previous screen by including a much larger dataset of cell lines and types (469 cell lines compared to 1 in the previous study). Additionally, we examined the effect of individual SLC expression, which severe knockout phenotypes might mask. By disregarding structural considerations in our analysis, we allow for the emergence of unexpected drug associations. However, this approach might lead to predicted drug associations not directly related to SLC transport of the drug itself, such as the metabolic environment of the cell and downstream events of SLC activity. Nevertheless, our analysis could aid in characterizing unknown SLC-drug interactions. The potential for polymorphisms in SLCs within human populations has been demonstrated to be a promising angle for personalized medicine [51]; our drug interaction results potentially extend this to include differences in expression as a method to predict the sensitivity of specific tumors to particular drugs in personalized medicine.

## Methods

### Workflow overview

**Fig. 6:**
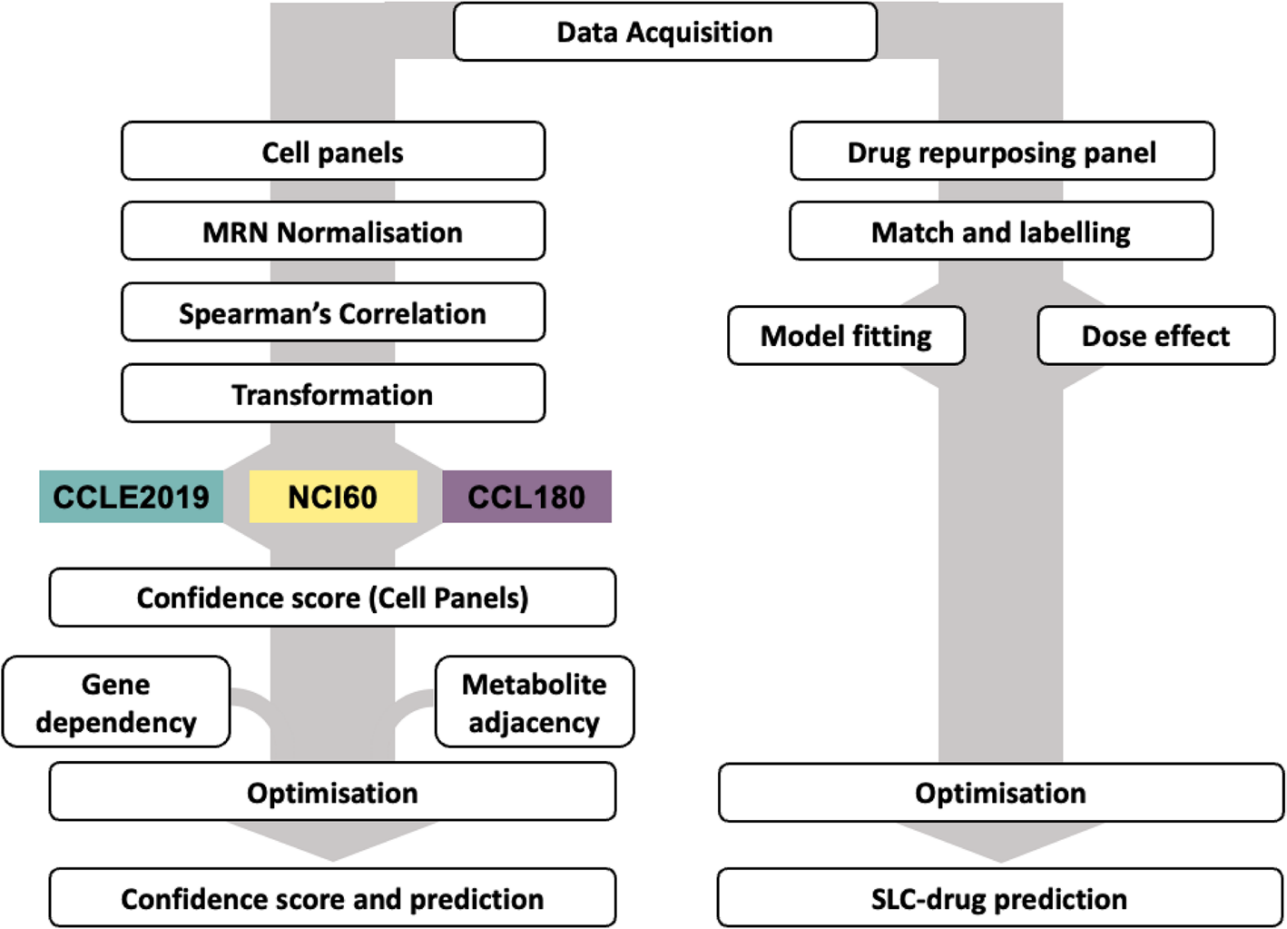
Overview of workflow. Illustration of the workflow used for the predictions described in detail in the methods section

Here we present an overview of the workflow, with details of each section described in the following sections (Fig. 6).

### Data acquisition for CCLE2019 dataset

CCLE2019 RNA-Seq and metabolomics data were downloaded from the DepMap Portal (depmap.org, CCLE 2019 omics) as read counts (file “CCLE_RNAseq_genes_counts_20180929.gct.gz”) and mean concentration levels (file “CCLE_metabolomics_20190502.csv”).

### Data acquisition for NCI60 dataset

NCI60 RNA-Seq data was derived from alignment and normalisation as performed previously [52]. NCI-60 metabolomics data were downloaded from the NCI DTP Data Portal (wiki.nci.nih.gov) as mean concentration levels (file “WEB_DATA_METABOLON.ZIP”).

### Data acquisition for CCL180 dataset

CCL180 metabolomics data were downloaded from the ETH Research Data Collection (https://doi.org/10.3929/ethz-b-000511784) as concentration levels and annotations (file “primary analysis (metabolomics)”).

### Data acquisition for CRISPR-Cas9 dependency screen

CRISPR-Cas9 dependency screen dataset was downloaded from DepMap portal (depmap.org, DepMap Public 23Q2 omics) as gene effect scores (file “CRISPRGeneEffect.csv”).

### Data acquisition for drug repurposing screen

Drug repurposing screen dataset was downloaded from Cancer Dependency Map Portal (https://depmap.org/repurposing), including cell line annotation (file “secondary-screen-cell-line-info.csv”), treatment metadata (“secondary-screen-replicate-collapsed-treatment-info.csv”) and viability log-fold (“secondary-screen-replicate-collapsed-logfold-change.csv”).

### Normalisation, correlation and transformation of SLC-metabolite pairs

Prior to all analysis, RNA-Seq read counts were normalised with Median Ratio Normalisation (MRN) by ‘DESeq2’ package in R to account for gene expression difference across different tissue types and cancer cell lines. Normalisation was applied to both CCLE 2019 and NCI60 raw counts across all cell lines. The data was first converted to a DESeqDataSet (dds) object using the ‘DESeqDataSetFromMatrix()’ function, and the sum of gene reads in each cell line was calculated and filtered if lower than 10. The resulting *dds* object was normalised by applying ‘estimateSizeFactors()’ function, and the normalised pseudocounts were extracted by ‘counts()’ function with argument ‘normalized = TRUE’. All subsequent analyses used the resulting normalised pseudocounts.

Correlation analysis was applied between CCLE 2019 pseudocounts and CCLE 2019 metabolomics (“CCLE2019”), CCLE 2019 pseudocounts and CCL180 metabolomics (“CCL180”), NCI-60 pseudocounts and NCI-60 metabolomics (“NCI60”). Spearman’s correlations were computed across mutually overlapping cell lines between pseudocounts and metabolite levels using the ‘cor.test()’ function in R with argument ‘method = “spearman”’.

The resulting correlation *p*-values were adjusted for each gene using Benjamini–Hochberg Procedure using the ‘p.adjust()’ function with ‘method = “BH”’. Correlation coefficients (ρ) might not be able to present correlation strength accurately across datasets due to differences in the distribution of correlation coefficients between different metabolites. Therefore, for ${SLC}_{a}$ and ${Metabolite}_{z}$, the transformed ρ coefficient $\widetilde{{\rho }_{(a,z)}}$ was computed using the following formula:

$$\widetilde{{\rho }_{(a,z)}}= \frac{\left|{\rho }_{(a,z)}\right|- \stackrel{-}{\left|{\rho }_{(z)}\right|}}{{\sigma }_{\left|{\rho }_{(z)}\right|}}$$

And thus represents the relative correlation strength of the pair.

### Concordance assessments

Between datasets, only mutually overlapping SLC and metabolite terms were assessed. The resulting raw ρ values for each overlapping SLC and metabolite were taken and correlated using the ‘cor.test()’ function in R with argument ‘method = “spearman”’.

### Benchmarking SLC-metabolite pairs

Known pair tables (Table S5, S11) were manually extracted from the SLC ontology annotation (Table S1) based on overlapping SLC and metabolite terms across datasets. Metabolites or drug molecules listed in the known pair tables were shuffled and randomly assigned to SLCs that are not known to transport them, while keeping the SLC column unchanged, resulting in 100 simulated random pair tables.

### CRISPR-Cas9 gene dependency screen analysis

CRISPR gene effect data recorded the gene effect score of 17,931 genes across 1095 cell lines of which there are 625 overlapping cell lines with CCLE2019 metabolomics, and 500 genes were annotated to be SLC or SLC-like. In the regard of CCLE2019 metabolomics, we transformed the metabolite levels in CCLE2019 metabolomics to Z-score by tissue to remove the impact from metabolite levels that are tissue-specific. For metabolite level ${Metabolite}_{i}$ in ${Tissue}_{j}$, the transformed metabolite level $\widetilde{{M}_{(i,j)}}$ was computed using the following formula:

$$\widetilde{{M}_{(i,j)}}= \frac{{M}_{(i,j)}-\overline{{M }_{j}}}{{\sigma }_{{M}_{j}}}$$

For analysis, we removed cell lines with positive gene effect scores in each SLC, and ranked the cell lines with gene effect score in ascending order. The highest and lowest 20% of cell lines were matched to CCLE2019 metabolomics, and the levels of every metabolite in these cell lines were compared using Wilcoxon’s test, with *p*-value adjusted for multiple comparison. The analysis resulted in a table contain SLC-metabolite pairs that discuss the possible metabolite difference when the particular SLC is targeted.

### Metabolite adjacency

The metabolite adjacency table (Table S8) was generated from human KEGG pathways using the ‘MetaboSignal’ package in R [53]. Specifically, all human metabolic pathways were extracted and subsetted using ‘MS_getPathIds()’ function with argument ‘organism_code = “hsa”’. The reaction network was built based on metabolic pathways using ‘MS_reactionNetwork()’ function. Subsequently, node distances were calculated using ‘MS_nodeBW()’ function with argument ‘node = “out”’ and ‘normalized = TRUE’.

### Confidence score

Before the addition of CRISPR loss-of-function screen and metabolite adjacency, for any pair $i$ (${SLC}_{i}-{Metabolite}_{i}$), the confidence score was computed as follow:

$${Confidence}_{i}= a \cdot ({CCLE2019}_{i} + {NCI60}_{i}+{CCL180}_{i})$$

where ${CCLE2019}_{i}$, ${NCI60}_{i}$, ${CCL180}_{i}$ are the sub-scores rewarded for performance of pair $i$ in each dataset; $a$ is the weight parameter for correlation outcomes (see Method Optimisation).

When CRISPR loss-of-function screen and metabolite adjacency were added, for any pair $i$ (${SLC}_{i}-{Metabolite}_{i}$), the confidence score is computed as follows:

$${Confidence}_{i}= a \cdot ({CCLE2019}_{i} + {NCI60}_{i}+{CCL180}_{i}) + b \cdot {GeneDep}_{i}+ c \cdot {Adjacency}_{i}$$

where ${GeneDep}_{i}$, and ${Adjacency}_{i}$ are the sub-scores rewarded for performance of pair $i$ in gene dependency analysis and metabolite adjacency; $b$ and $c$ are optimised weight parameters (see Method Optimisation) used to add weight to sub-scores.

The sub-score in each category is calculated based on its respective threshold of discovery ($ToD$, derived as optimised, see method Optimisation), and how well the value performance (${V}_{i}$) is compared to the distribution of values in the known set (${V}_{k}$). If ${V}_{i}<ToD$, a sub-score of 0 is assigned. If ${V}_{i} \ge ToD$, ${V}_{i}$ is compared to the distribution of ${V}_{k}$ for score assignment. Specifically, a set of quantiles (${Q}_{k}$) are derived from the distribution of ${V}_{k}$ from the 0th percentile (minimum, ${q}_{k0}$) to the 100th percentile (maximum, ${q}_{k10}$), with increments of 10% and the minimum of ${V}_{k}$ (${q}_{k0}$) being set to the value of $ToD$:

$${Q}_{k}=\left\{{q}_{k0},{q}_{k1}, {q}_{k2}, {q}_{k3},\dots ,{q}_{k10}\right\}, {q}_{k0}=ToD$$

Next, we find the index of the interval ($j$) in the array ${Q}_{k}$ where ${V}_{i}$ would fall (i.e. $j$ is taken when the condition ${q}_{kj-1}\le {V}_{i}<{q}_{kj}$ holds true), and assigned it as sub-score:

$${Subscore}_{i}= j | {q}_{kj-1}\le {V}_{i}<{q}_{kj}$$

As a result, values in ${Q}_{k}$ will be assigned a score between 1 (${q}_{k0}\le {V}_{i}<{q}_{k1}$), and 11 (${V}_{i}={q}_{k10}$, which satisfies the condition ${q}_{k10}\le {V}_{i}<{q}_{k11}$). Since ${Q}_{k}$ only represents the distribution of known set, any ${V}_{i}$ outside of the known set and satisfies ${V}_{i}\ge {q}_{k10}$ will be capped at 11. Subsequently, ${Subscore}_{i}$ will be an integer ranging from 0 to 11, according to which interval the value in this category falls. In R, ${Q}_{k}$ is derived by ‘quantile()’ with argument ‘probs = seq(0, 1, 0.1)’, and ${Subscore}_{i}$ is derived by ‘findInterval()’.

For analysis of correlation outcomes in cell panels and metabolite adjacency, ${V}_{i}$ represents the transformed ρ for pair $i$ in the respective set, and ${Q}_{k}$ represents the collection of transformed ρ for pairs in known pairs table. For the gene dependency screen, ${V}_{i}$ represents the log_10_-transformed Wilcoxon’s adjusted *p*-value for pair $i$, and ${Q}_{k}$ represents the collection of log_10_-transformed Wilcoxon’s adjusted *p*-value for pairs in known pairs table.

For any pair $i$ (${SLC}_{i}-{Metabolite}_{i}$), ${Adjacency}_{i}$ is calculated as the sum of sub-scores of all the correlations from ${SLC}_{i}$ to metabolite that’s adjacent (i.e. within 2 units of adjacency) to ${Metabolite}_{i}$ and better than the $ToD$ (adjacent metabolites to ${Metabolite}_{i}$ are denoted as ${Adj({M}_{i})}_{1}, {Adj({M}_{i})}_{2}, \dots , {Adj({M}_{i})}_{n}$):

$${Adjacency}_{i}= \sum_{n=1}^{n}Subscore({Adj({M}_{i})}_{n})$$

where sub-score is calculated based on $ToD$ in cell panels ($ToD$) and in adjacency (${ToD}{\prime}$) that’s been optimised beforehand (see Method Optimisation):

$${Q}_{k}=\{{q}_{k0},{q}_{k1}, {q}_{k2}, {q}_{k3},\dots ,{q}_{k10}\}, {q}_{k0}=ToD$$

$${Subscore}_{i}= \left\{\begin{array}{c}0, if {V}_{i}<{ToD}{\prime}\\ 0.5, if {ToD}{\prime}< {V}_{i}\le ToD \\ j | {q}_{kj-1}\le {V}_{i}<{q}_{j}, if {V}_{i}>ToD\end{array}\right.$$

Take a pair, ${SLC}_{i}-Glutamate$ (any SLC to compound glutamate) as an example. The compound glutamate (KEGG ID: C00025) can link to 70 metabolites in the adjacency matrix, and 10 of them are within 2 steps of conversion and were measured in at least one metabolomics in cell panels. Therefore, 10 sub-scores will be derived from the 10 correlations between ${SLC}_{i}$ and 10 metabolites adjacent to glutamate, and $Adjacency$ for the pair ${SLC}_{i}-Glutamate$ is calculated as the sum of these 10 sub-scores. We took caution to calculate adjacency for molecules that participate in extremely large number of reactions (such as ATP), and we made sure only direct conversions in their biosynthesis pathway were considered.

### Optimisation

The value of parameters in the confidence score calculation will affect the final score calculated for pairs. For any given value of parameter, we generated the final score, and surveyed the score distribution as we recorded the fraction of pairs left when we increase the score cutoffs (i.e. only pairs with scores higher than the score cutoff will be regarded as confident pairs). We define a parameter is optmised if the given parameter maximised the mean fractional difference across cutoff between score assigned to pairs in known set (fraction of true positive) and in simulated set by benchmarking (fraction of false positive).

In cell panel (NCI60, CCLE2019, CCL180), the parameter $a$ was given the value 3, and values of $ToD$ were optimised in each dataset. A $ToD$ of 0 means the pair (${SLC}_{i}-{Metabolite}_{i}$) will only be considered for sub-score calculation if the pair has a transformed ρ higher than 0 (i.e. the mean of absolute raw ρ of all SLC correlating to ${Metabolite}_{i}$); A $ToD$ of 1.0 means the threshold of consideration is increased to a standard deviation higher than mean. $ToD$ and weight parameter $b$ of CRISPR, ${ToD}{\prime}$ and weight parameter $c$ of Adjacency were optimised individually following the same process. Table 1 demonstrates the parameter we used to calculate the final confidence score:

**Table 1:** Confidence score parameters

$Dataset$	$ToD$	$Parameter$	${ToD}{\prime}$	$Parameter$
$NCI60$	$0.0$	$a=3.0$	$0.1$	$c=1.0$
$CCLE2019$	$0.0$		$0.1$	
$CCL180$	$1.0$		$0.2$	
$CRISPR$	$0.16$	$b=1.0$	$/$	$/$

Parameters as described in the text are indicated for the different datasets

Here, we present an example pair, SLC35B1 – glutamate, of how confidence score was calculated in full (Table 2).

**Table 2:** Confidence score calculation for SLC35B1 and glutamate (Cell panels and gene dependency

	$NCI60$	$CCLE2019$	$CCL180$	$GeneDep (-{log}_{10})$
$Transformed \, \rho$	$-0.97$	$0.96$	$0.019$	$9.7 \times {10}^{-5}(4.01)$
$Percentile$	${V}_{i}< ToD (0.0)$	$50\%\le {V}_{i}< 60\%$	${V}_{i}< ToD (1.0)$	$70\%\le {V}_{i}< 80\%$
$Score$	$0$	$6$	$0$	$8$
$Weighted\ Score$	$0$	$6 \times 3=18$	$0$	$8\times 1=8$
$Confidence\ Score$	$0 +18+0=18$			$8$

Example confidence score calculation demonstrating the final output scores.

Calculating from cell panels, the pair SLC35B1 – glutamate has a confidence score of 18, inclusion of gene dependency will give the pair the total score of 26. Glutamate has 10 derivatives found within 2 steps of conversions, of which 2 fall in $80\%\le {V}_{i}< 90\%$ in NCI60; 2 fall in $80\%\le {V}_{i}< 90\%$ in CCLE2019; 1 falls in ${ToD}{\prime}\left(0.2\right)< {V}_{i}\le ToD(1.0)$, and 1 falls in $90\%\le {V}_{i}< 100\%$ in CCL180. All the rest are either not measured in the respective dataset, or ${V}_{i}<{ToD}{\prime}$. As a result, the adjacency score will be:

$$\left[\left(2 \times 9\right)+\left(2 \times 9\right)+\left(0.5+ 10\right)\right]=46.5$$

The final confidence score therefore is the sum of cell panels (18), gene dependency (8), and metabolite adjacency (46.5), resulting 72.5.

### Partitioning, training and testing

In order to further test how our algorithm performed we partitioned 667 pairs in the known pair table (Table S5) randomly into a training set (466/667, 70% of all pairs) and a testing set (201/667, 30% of all pairs). We performed optimisation on the training set, and report the confidence score calculated with optimised parameters in the testing set. This process was repeated for 10 iterations.

### Drug prediction algorithm

The drug repurposing panel records 1448 active drugs in 8 doses against 581 cell lines, of which 569 overlap with CCLE2019 transcriptomics. To interrogate the dose response between ${SLC}_{i}$ and ${Drug}_{i}$, we ranked the cell lines in the CCLE2019 transcriptomics dataset according to the expression level of ${SLC}_{i}$ in descending order, and labelled the first and last 20% of cell lines as “high expression” and “low expression” accordingly. The labelled cell lines were matched back to the drug repurposing panel to extract the cellular viability post ${Drug}_{i}$ treatment in 8 log_10_ transformed doses (−3.21, −2.61, −2.01, −1.40, −0.81, −0.20, 0.40, 1.00). We captured the shape information of the dose response, as we fitted local polynomial regression models to the two curves (“high expression” and “low expression”), and predicted 421 data points from −3.21 to 1.00, incremented by 0.01 with ‘predict()’ function. The curves were compared with a paired t-test. The resulting *p*-value and mean differences were recorded.

We assessed the dose effect by calculating the pair-wise difference between predicted data points of the two curves, and correlated the difference with the dose using Spearman’s correlation. The resulted *p*-value and absolute Spearman’s ρ were recorded.

To yield predictions, we calculated two drug-specific significance thresholds. For every ${Drug}_{i}$, we randomly picked 20% of the cell lines (93) and recorded their resulted *p*-value and mean differences. The resulting 100 *p*-values were -log_10_ transformed (-log_10_ transformed *p*-value), and the difference between absolute values of the two were calculated (absolute mean difference). We derived the standard deviation and mean from both -log_10_ transformed *p*-value and absolute mean difference, and set them as the null expectation. For any pair ${SLC}_{i}-{Drug}_{i}$, only when both its -log_10_(*p*-value) and absolute mean differences are higher than the null expectation, will it be considered as “predicted”.

### ROC and PR AUC

In this study, predictive power of the algorithm or predictors were measured with Area-under-curve (AUC) of Receiver-operating characteristic (ROC) and Precision-Recall (PR) curves. For both ROC and PR curves, AUC was calculated following the trapezoidal rule.

### Code availability

Primary data for this paper is all published and can be accessed via links in the Materials and Methods sections. All results are in the supplemental tables. Code required to produce the figures and the code required to process the primary data is available on GitHub: https://github.com/SarkiesLab/DeorphanizeSLCs.

## Supplementary Information

Supplementary Material 1.

Supplementary Material 2.

Supplementary Material 3.

Supplementary Material 4.

Supplementary Material 5.

Supplementary Material 6.

Supplementary Material 7.

Supplementary Material 8.

Supplementary Material 9.

Supplementary Material 10.

Supplementary Material 11.

Supplementary Material 12.

Supplementary Material 13.

Supplementary Material 14.

Supplementary Material 15.

Supplementary Material 16.

Supplementary Material 17.

Supplementary Material 18.

Supplementary Material 19.

Supplementary Material 20.

## Data Availability

Primary data for this paper is all published and can be accessed via links in the Materials and Methods sections.  All results are in the supplemental tables.  Code required to produce the figures and the code required to process the primary data is available on GitHub: https://github.com/SarkiesLab/DeorphanizeSLCs.
