# Supplementary figures and images for: Predicting substrates for orphan solute carrier proteins using multi-omics datasets
Source: BMC Genomics. 2025 Feb 11;26:130. doi: 10.1186/s12864-025-11330-5 (PMC11812203; doi:10.1186/s12864-025-11330-5)

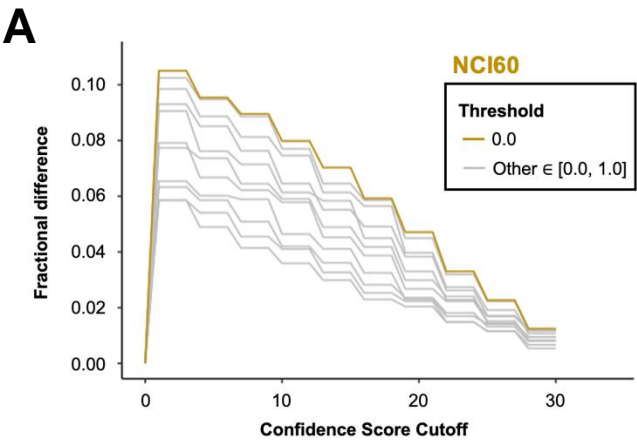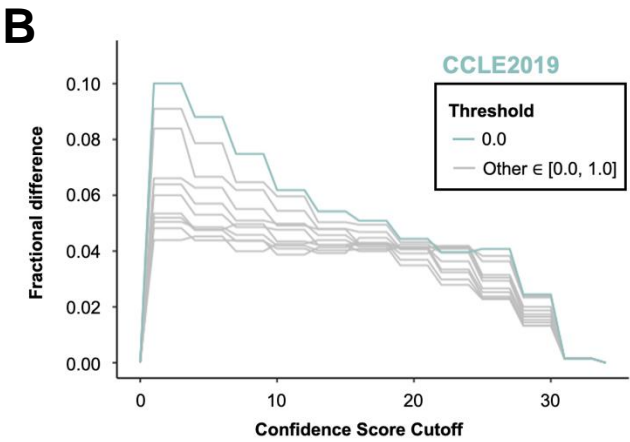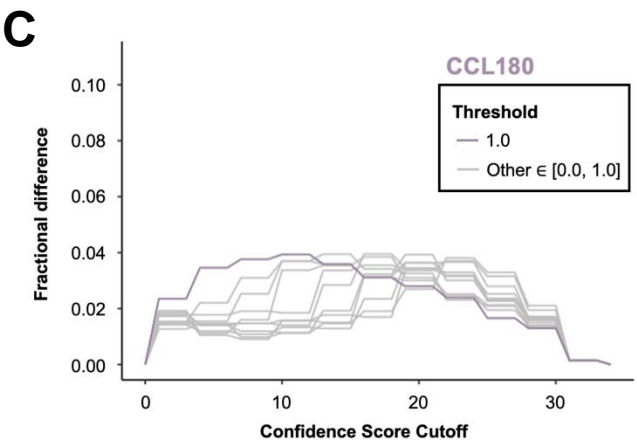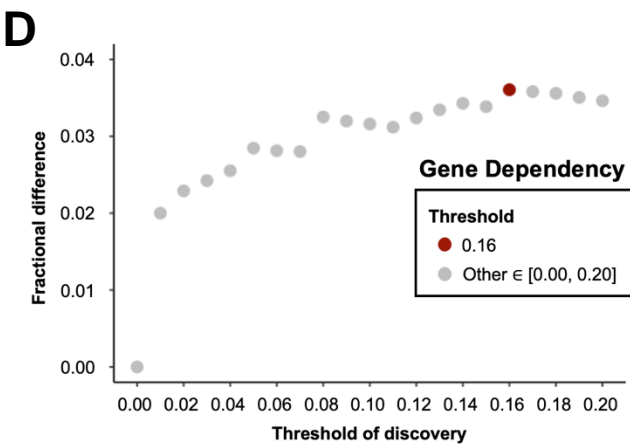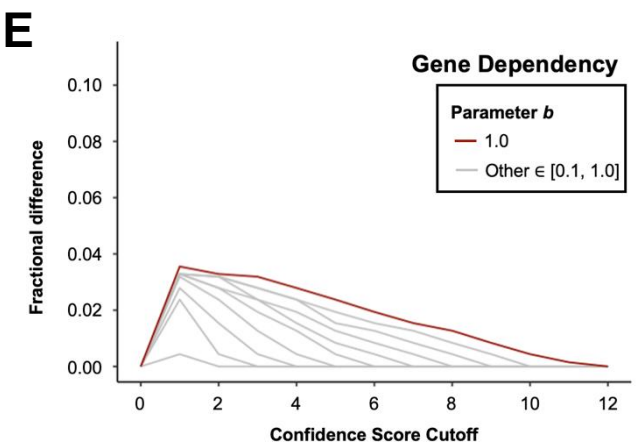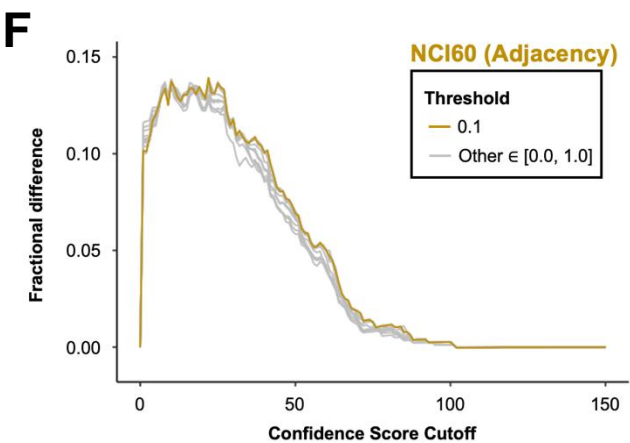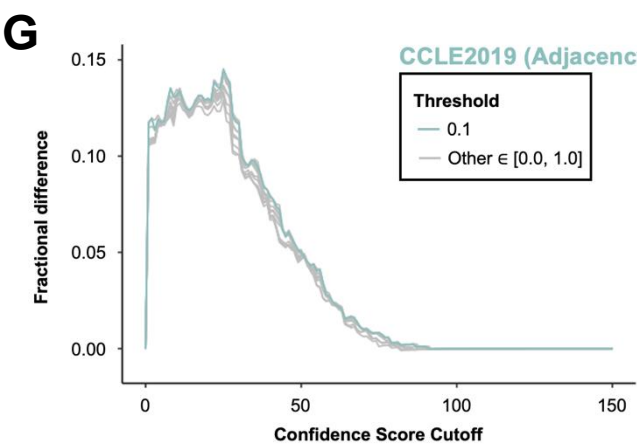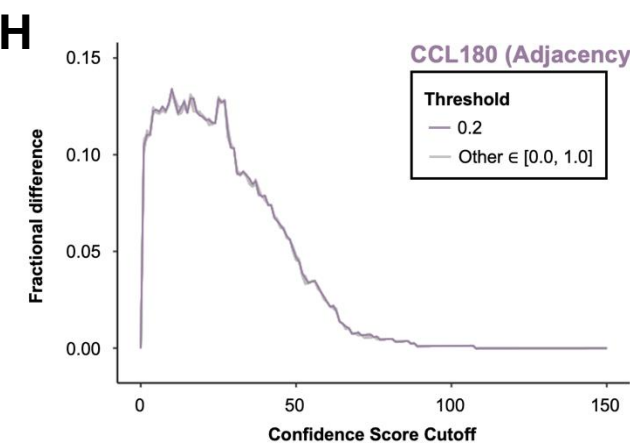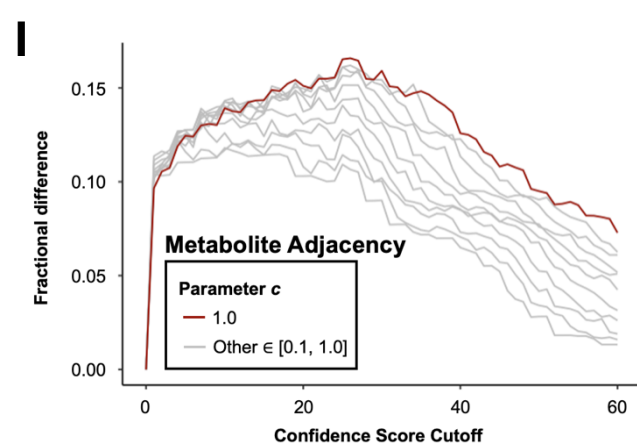

Figure S1

Supplement: Supplementary file 1 — Supplementary Material 1. [file 12864_2025_11330_MOESM1_ESM.pdf]

**A**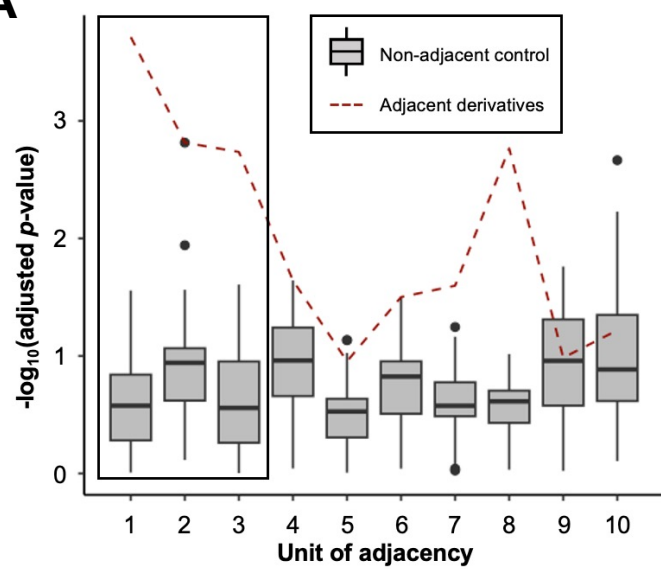**B**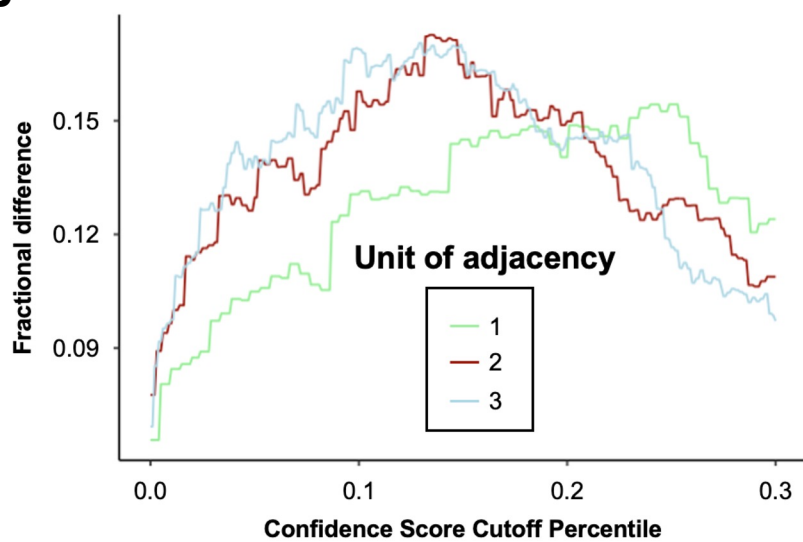**Figure S2**

Adjacency transformed rho

Supplement: Supplementary file 2 — Supplementary Material 2. [file 12864_2025_11330_MOESM2_ESM.pdf]

**A**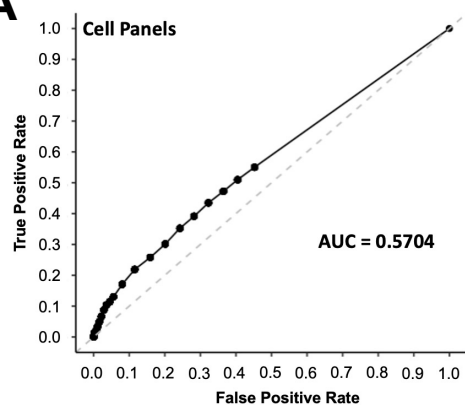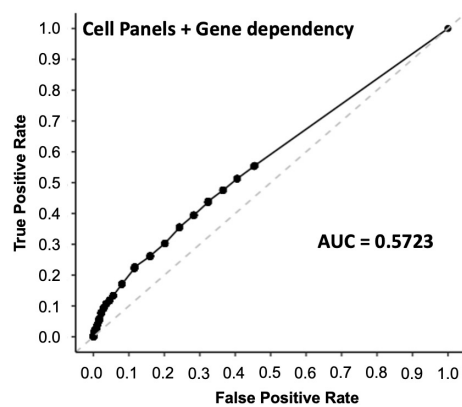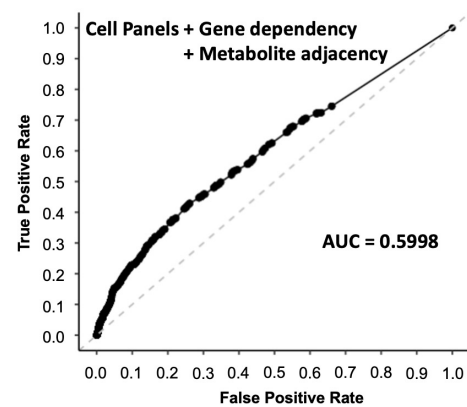**B**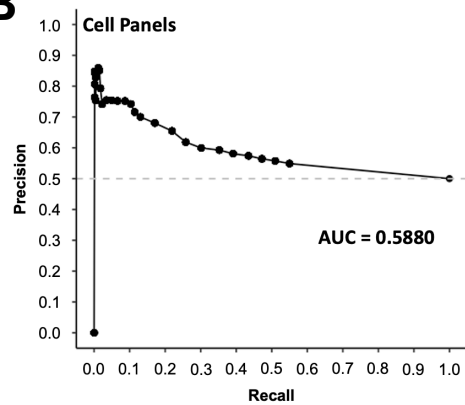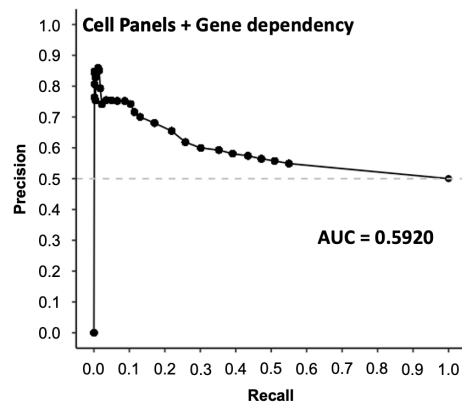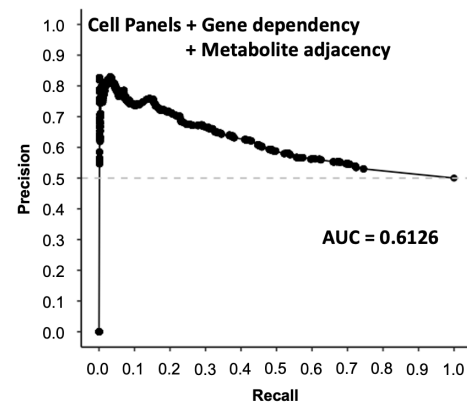**C**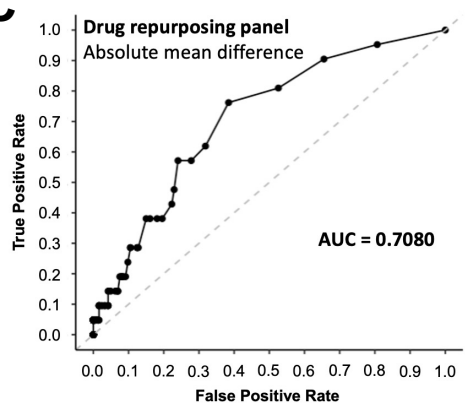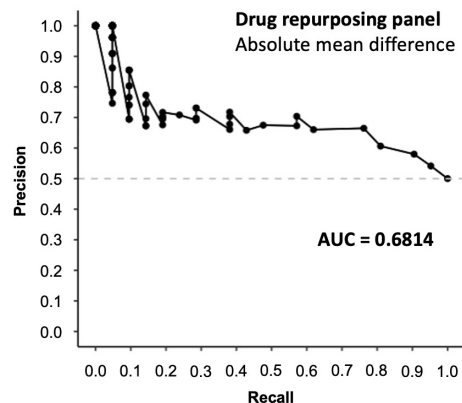**D**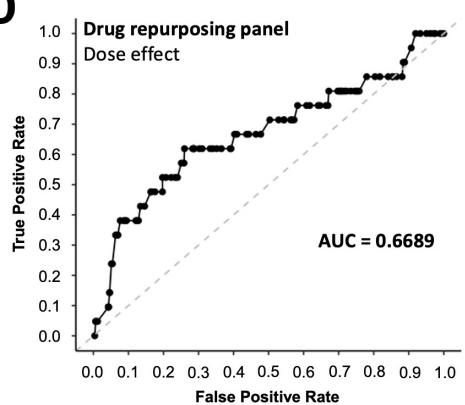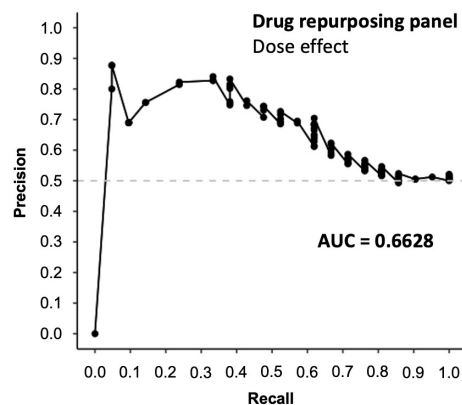**Figure S3**

Supplement: Supplementary file 3 — Supplementary Material 3. [file 12864_2025_11330_MOESM3_ESM.pdf]

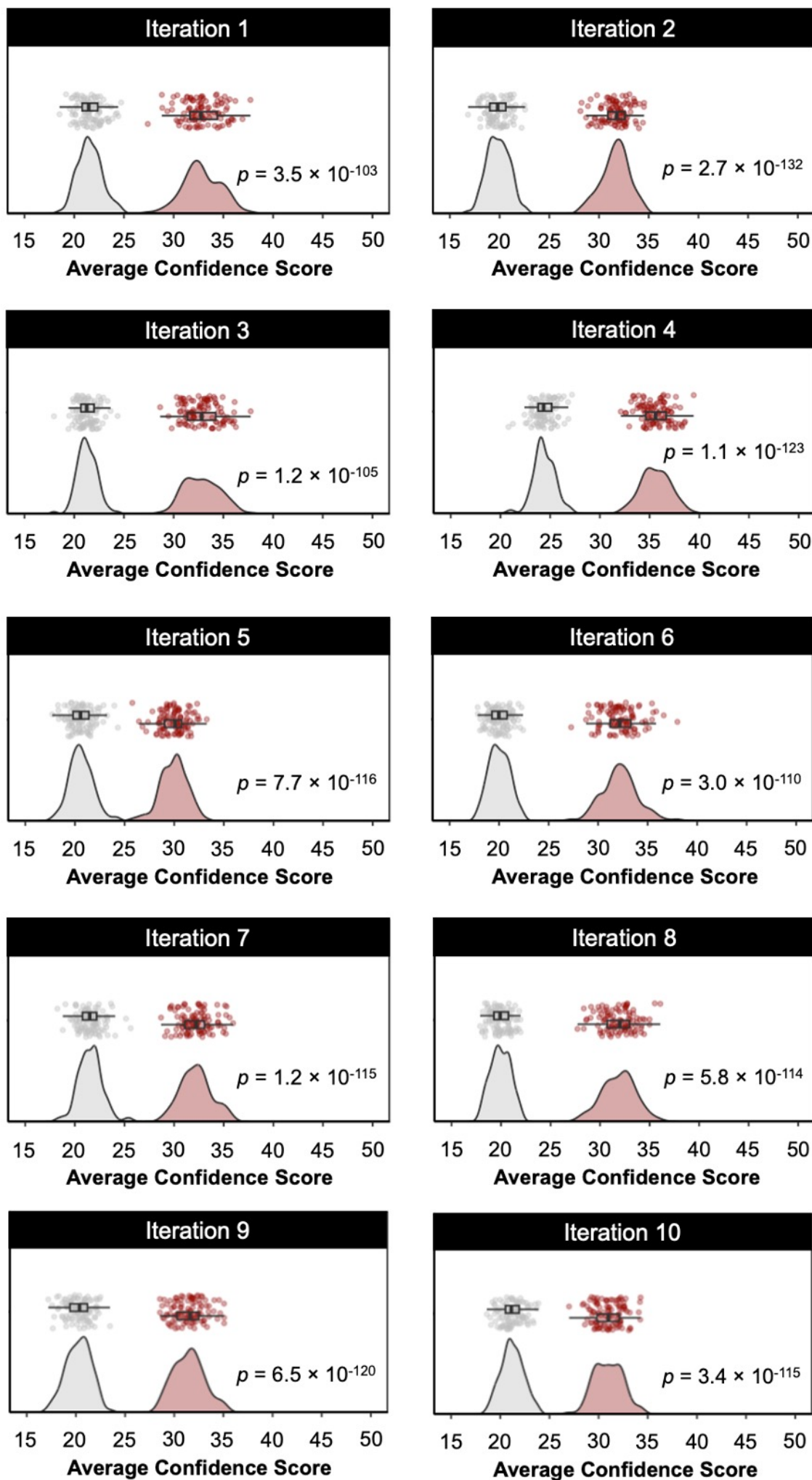

Figure S4

Supplement: Supplementary file 4 — Supplementary Material 4. [file 12864_2025_11330_MOESM4_ESM.pdf]

**A**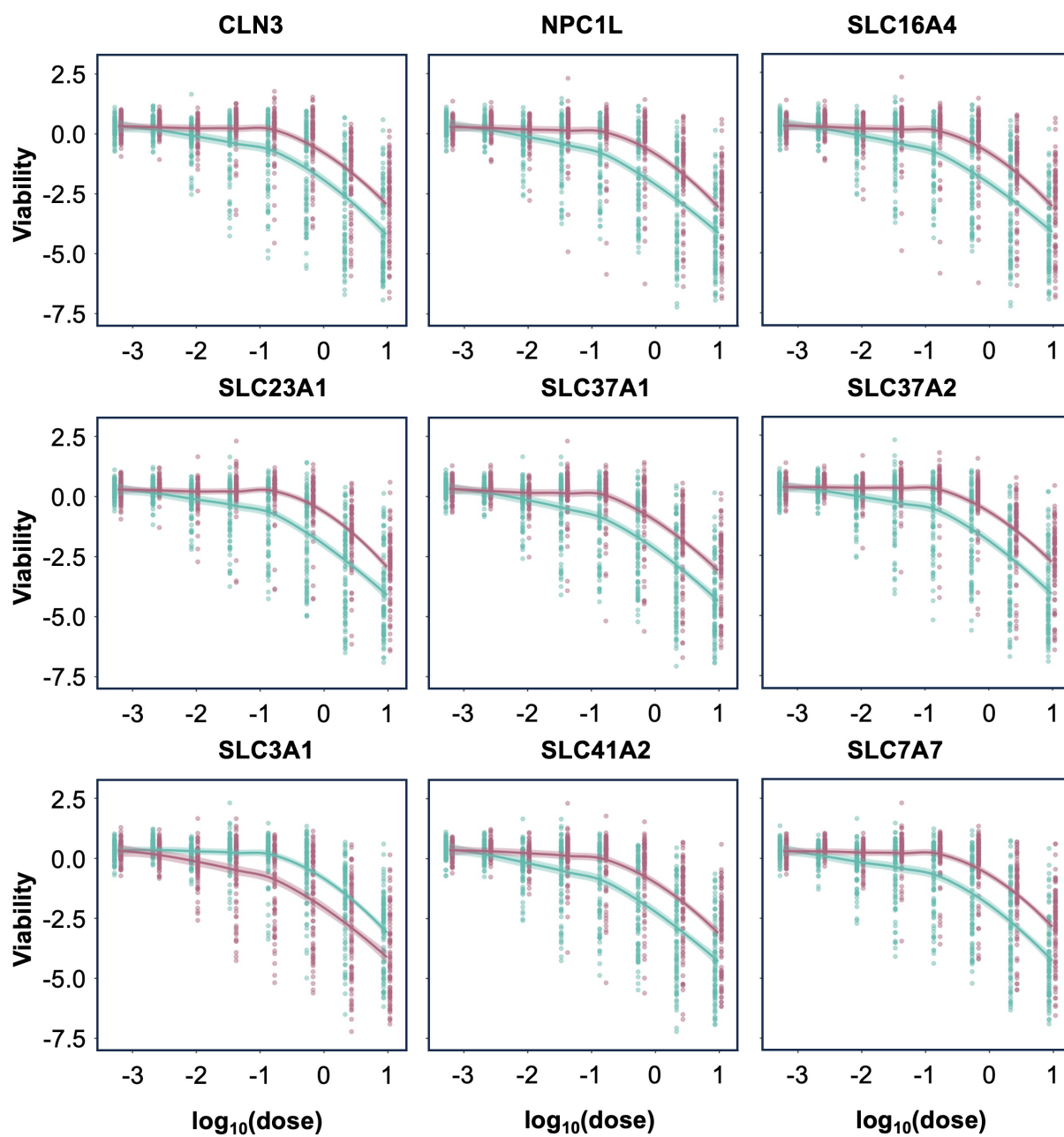**B**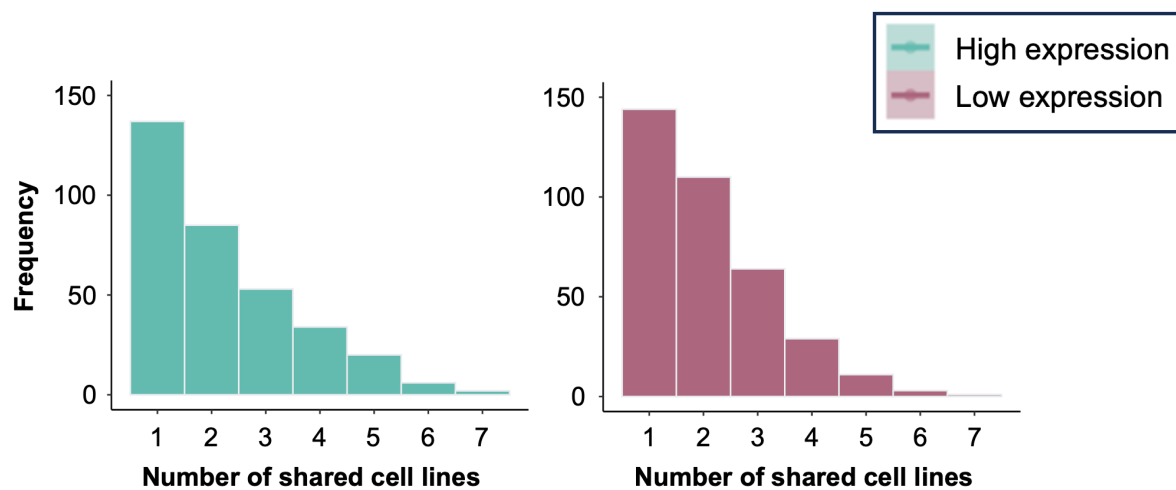**Figure S5**

Supplement: Supplementary file 5 — Supplementary Material 5. [file 12864_2025_11330_MOESM5_ESM.pdf]
